# Neoadjuvant chemotherapy plus intensity-modulated radiotherapy versus concurrent chemoradiotherapy plus adjuvant chemotherapy for the treatment of locoregionally advanced nasopharyngeal carcinoma: a retrospective controlled study

**DOI:** 10.1186/s40880-015-0076-9

**Published:** 2016-01-06

**Authors:** Wen-Ze Qiu, Pei-Yu Huang, Jun-Li Shi, Hai-Qun Xia, Chong Zhao, Ka-Jia Cao

**Affiliations:** Department of Nasopharyngeal Carcinoma, State Key Laboratory of Oncology in South China, Collaborative Innovation Center of Cancer Medicine, Sun Yat-sen University Cancer Center, Guangzhou, Guangdong 510060 P.R. China; Department of Radiation Oncology, The Second Hospital of Tianjin Medical University, Tianjin, 300211 P.R. China

**Keywords:** Nasopharyngeal carcinoma, Intensity-modulated radiotherapy, Neoadjuvant chemotherapy, Concurrent chemoradiotherapy, Adjuvant chemotherapy

## Abstract

**Background:**

In the era of intensity-modulated radiotherapy (IMRT), the role of neoadjuvant chemotherapy (NAC) for locoregionally advanced nasopharyngeal carcinoma (NPC) is under-evaluated. The aim of this study was to compare the efficacy of NAC plus IMRT and concurrent chemoradiotherapy (CCRT) plus adjuvant chemotherapy (AC) on locoregionally advanced NPC.

**Methods:**

Between January 2004 and December 2008, 240 cases of locoregionally advanced NPC confirmed by pathologic assessment in Sun Yat-sen University Cancer Center were reviewed. Of the 240 patients, 117 received NAC followed by IMRT, and 123 were treated with CCRT plus AC. The NAC + IMRT group received a regimen that included cisplatin and 5-fluorouracil (5-FU). The CCRT + AC group received cisplatin concurrently with radiotherapy, and subsequently received adjuvant cisplatin and 5-FU. The survival rates were assessed by Kaplan–Meier analysis, and the survival curves were compared using a log-rank test. Multivariate analysis was conducted using the Cox proportional hazard regression model.

**Results:**

The 5-year overall survival (OS), locoregional relapse-free survival (LRRFS), distant metastasis-free survival (DMFS), and disease-free survival (DFS) were 78.0, 87.9, 79.0, and 69.8%, respectively, for the NAC + IMRT group and 78.7, 84.8, 76.2, and 65.6%, respectively, for the CCRT + AC group. There were no significant differences in survival between the two groups. In multivariate analysis, age (<50 years vs. ≥50 years) and overall stage (III vs. IV) were found to be independent predictors for OS and DFS; furthermore, the overall stage was a significant prognostic factor for DMFS. Compared with the CCRT + AC protocol, the NAC + IMRT protocol significantly reduced the occurrence rates of grade 3–4 nausea–vomiting (6.5 vs. 1.5%, *P* = 0.023) and leukopenia (9.7 vs. 0.8%, *P* = 0.006).

**Conclusions:**

The treatment outcomes of the NAC + IMRT and CCRT + AC groups were similar. Distant metastasis remained the predominant mode of treatment failure.

## Background

Nasopharyngeal carcinoma (NPC) is endemic in South China and Southeast Asia, with an annual incidence of 15–50 cases per 100,000 [1]. NPC mortality in China, especially in South China, was also at high levels [2]. Radiotherapy is the primary treatment modality for NPC, and the outcome for patients with early-stage disease is usually favorable; however, the response of locoregionally advanced NPC to radiotherapy is unsatisfactory [3].

Combining chemotherapy and radiotherapy is a reasonable strategy for improving the long-term outcome of locoregionally advanced NPC. The Intergroup 0099 Study (IGS) was the first randomized, controlled trial to achieve a significant improvement in the 3-year overall survival (OS) for stages III-IVb NPC patients by adding concurrent and adjuvant chemotherapy to conventional radiotherapy [4]. Since then, this regimen has been deemed the standard of care for advanced NPC. However, there have been serious concerns regarding the applicability of the IGS results, and the outcomes of several randomized, controlled trials attempting to verify the efficacy of concurrent chemoradiotherapy (CCRT) plus adjuvant chemotherapy (AC) were conflicting [5–9]. With the advent of intensity-modulated radiotherapy (IMRT), local control has been substantially improved, and distant metastasis is now the main cause of treatment failure [10]. Further improvements in systemic control by the use of concurrent chemotherapy is unlikely because of drug-related toxic effects [7]; data from several published studies [11, 12] show that AC does not translate to significant improvements in prolonging OS and reducing distant metastatic rate (DMR). One strategy for treatment improvement is to change the sequence from concurrent-adjuvant to addition of neoadjuvant chemotherapy (NAC) prior to radiotherapy. It is important to address the efficacy of NAC because meta-analyses showed that NAC could significantly reduce both locoregional and distant failures [13, 14]. A pooled meta-analysis of two trials by Chua et al. [13] noted that modest improvements in relapse-free survival and disease-specific survival could be achieved by NAC. Another meta-analysis by Ouyang et al. [14] revealed significant treatment efficacy in terms of OS and DMR for NAC.

Because most of these studies were based on the non-IMRT technique, the role of NAC plus IMRT for locoregionally advanced NPC in the era of IMRT is unknown. The aim of this study was to compare the efficacy of NAC plus IMRT with CCRT plus AC on locoregionally advanced NPC.

## Methods

### Patients

Inclusion criteria were as follows: pathologically diagnosed non-keratinizing or undifferentiated carcinoma of the nasopharynx (World Health Organization [WHO] type II or III); age of 18–65 years; stages III-IVb disease according to the 2002 Union for International Cancer Control (UICC) Staging System; no evidence of distant metastasis; Karnofsky performance score ≥70; normal hematologic function [white blood cell (WBC) count ≥4.0 × 10^9^/L, platelet (PLT) count ≥100 × 10^9^/L]; normal hepatic function [total bilirubin (TBIL) and alanine aminotransferase (ALT) <2 times the normal values]; normal renal function [creatinine (Cr) <1.5 times the normal value]; receiving radical IMRT at initial diagnosis; and receiving NAC with cisplatin and 5-fluorouracil (5-FU) for two cycles or receiving concurrent chemotherapy with cisplatin for two cycles plus AC with cisplatin and 5-FU for two cycles.

### Radiotherapy

All patients were immobilized in the supine position with a head, neck, and shoulder thermoplastic mask. Two sets of images, with and without contrast, were obtained from the computed tomography (CT) simulator for treatment planning. All patients were scanned with serial 3-mm slices from the vertex through the clavicles. Inverse IMRT planning was performed using the Corvus system, version 3.0 (Peacock, Nomos, Deer Park, IL, USA), and a dynamic, multileaf, intensity-modulating collimator (MIMiC; Nomos Corp., Sewickly, PA, USA) was used for planning and treatment. The gross tumor volumes of the nasopharynx (GTVnx) and positive neck lymph nodes (GTVnd) were delineated according to our previously described institutional treatment protocol [15], which is in agreement with the International Commission on Radiation Units and Measurements Reports 50 [16] and Reports 62 [17]. The first clinical tumor volume (CTV1) was defined as the GTVnx plus a margin of 5–10 mm for potential microscopic spread, including the entire nasopharyngeal mucosa plus a 5-mm submucosal volume. The second CTV (CTV2) was defined by adding a margin of 5–10 mm to CTV1 and included the following regions, which needed prophylactic irradiation: retropharyngeal lymph node regions, the clivus, skull base, pterygoid fossae, parapharyngeal space, inferior sphenoid sinus, posterior edge of the nasal cavity, maxillary sinuses, and the lymphatic drainage area. The planning target volume (PTV) for GTVs and CTVs were generated automatically by adding a 5-mm margin after delineation of tumor targets according to the immobilization and localization uncertainties. The prescribed dose was 68–70 Gy to the PTV of the GTVnx (PTVnx), 64–68 Gy to the PTV of the GTVnd (PTVnd), 60 Gy to the PTV of the CTV1 (PTV1), and 54 Gy to the PTV of the CTV2 (PTV2) in 30 fractions. All patients were treated with one fraction daily over 5 days per week.

### Chemotherapy

For the NAC + IMRT group, two NAC cycles, consisting of cisplatin (80 mg/m^2^ intravenous infusion on day 1) and 5-FU (4 g/m^2^ daily as a 120-h intravenous infusion on days 1–5), were administered at an interval of 3 weeks before radiotherapy.

For the CCRT + AC group, two concurrent chemotherapy cycles, consisting of cisplatin (80 mg/m^2^ intravenous infusion on days 1 and 22), were administered at an interval of 3 weeks during radiotherapy; 2 subsequent AC cycles, consisting of cisplatin (80 mg/m^2^ intravenous infusion on day 1) and 5-FU (4 g/m^2^ daily as a 120-h intravenous infusion on days 1–5), were administered at an interval of 3 weeks after the completion of radiotherapy.

### Patient assessment and follow-up

Clinical evaluation of the efficacy of therapy was based on the Response Evaluation Criteria in Solid Tumors (RECIST) v1.1 [18]. Acute and late adverse events were assessed according to the Common Terminology Criteria for Adverse Events (CTCAE) v3.0 [19]. Patients were evaluated weekly during radiotherapy and every 3 months for the first 3 years, every 6 months for the fourth and fifth years, and every year thereafter. Follow-up evaluation included physical examination of the head and neck, nasopharyngeal endoscopy, chest radiography, abdominal ultrasound, Epstein–Barr virus (EBV) serological testing, and magnetic resonance imaging (MRI) or CT of the head and neck region. CT scans of the abdominopelvic cavity or chest, bone scans, and positron emission tomography scans were conducted when clinically indicated.

### Statistical analysis

The SPSS 16.0 statistical software (SPSS Inc., Chicago, IL, USA) was used. The Chi square analysis was used to compare occurrence rates of adverse events and categorical variables. The means of continuous variables were compared using the Student’s *t* test. The study endpoints of OS, LRRFS, DMFS, and DFS were determined by patient death, relapse of a local or nodal tumor, occurrence of distant metastasis, and occurrence of relapse or distant metastasis respectively. The time-to-event for each endpoint was calculated from the date of completion of treatment to the occurrence date of the event using the Kaplan–Meier method. Statistical differences in endpoints were estimated using the log-rank test. The multivariate analysis was conducted by the Cox proportional hazard regression model. A two-tailed *P* value of less than 0.05 was considered significant.

## Results

### Patient characteristics

Between January 2004 and December 2008, clinical data of 240 NPC patients treated in the Sun Yat-sen University Cancer Center who met all of the criteria were retrospectively analyzed. Of these patients, 175 were males and 65 were females. The median age was 44 years. Of the 240 patients, 117 received NAC followed by IMRT, and 123 were treated with CCRT plus AC. No significant differences were found between the two groups in baseline characteristics (Table 1).

**Table 1:** Characteristics of the 240 patients with locoregionally advanced nasopharyngeal carcinoma (NPC)

Characteristic	NAC + IMRT [cases (%)]	CCRT + AC [cases (%)]	χ^2^	***P***
Total	117	123		
Age (years)			0.199	0.656
<50	83 (70.9)	84 (68.3)		
≥50	34 (29.1)	39 (31.7)		
Gender			1.148	0.284
Male	89 (76.1)	86 (69.9)		
Female	28 (23.9)	37 (30.1)		
T stage			1.029	0.794
T1	4 (3.4)	4 (3.3)		
T2	11 (9.4)	14 (11.4)		
T3	57 (48.7)	65 (52.8)		
T4	45 (38.5)	40 (32.5)		
N stage			3.578	0.311
N0	22 (18.8)	27 (22.0)		
N1	40 (34.2)	50 (40.6)		
N2	46 (39.3)	42 (34.1)		
N3	9 (7.7)	4 (3.3)		
Clinical stage			2.256	0.133
III	65 (55.6)	80 (65.0)		
IV	52 (44.4)	43 (35.0)		
WHO histology			0.313	0.576
II	13 (11.1)	11 (8.9)		
III	104 (88.9)	112 (91.1)		

*NAC* neoadjuvant chemotherapy, *IMRT* intensity-modulated radiotherapy, *CCRT* concurrent chemoradiotherapy, *AC* adjuvant chemotherapy, *WHO* World Health Organization

### Treatment and compliance

All patients completed the full course of radiotherapy. In the NAC + IMRT group, all patients completed two cycles of NAC. In the CCRT + AC group, all patients completed two cycles of concurrent chemotherapy and two cycles of AC.

### Clinical response

After two cycles of NAC with cisplatin plus 5-FU, five patients (4.3%) had a complete response (CR), and 93 (79.5%) had a partial response (PR) in the NAC + IMRT group. Three months after radiotherapy, the effective rates (CR plus PR) were 98.3% (115/117) in the NAC + IMRT group and 98.4% (121/123) in the CCRT + AC group (*P* = 1.000).

### Patterns of treatment failure

The failure patterns, locoregional or distant, in the NAC + IMRT and CCRT + AC groups were similar (Table 2).

**Table 2:** Patterns of disease failure in patients treated with NAC plus IMRT vs. CCRT plus AC

Failure pattern	NAC + IMRT [cases (%)]	CCRT + AC [cases (%)]	*P*
Locoregional relapse only	11 (9.4)	12 (9.8)	0.926
Distant metastases only	22 (18.8)	26 (21.1)	0.651
Both locoregional relapse and distant metastases	2 (1.7)	6 (4.9)	0.314
Death	28 (23.9)	37 (30.1)	0.284

Abbreviations as in Table 1

### Acute and late toxicities

Acute toxicities during radiotherapy were well tolerated by the entire group. Compared with the CCRT + AC group, the NAC + IMRT group had significantly reduced occurrence rates of grades 3–4 nausea–vomiting (6.5 vs. 1.5%, *P* = 0.023) and leukopenia (9.7 vs. 0.8%, *P* = 0.006). No significant differences in anemia, thrombocytopenia, liver dysfunction, and mucositis were found between the two groups (Table 3). The most common late toxicities were xerostomia, hear loss, skin dystrophy, subcutaneous fibrosis, and temporal lobe injury (TLI). Ten patients (4.2%) developed TLI. Among them, 4 had radiation injuries in unilateral temporal lobes, and 6 in bilateral temporal lobes. No significant differences in late toxicities were found between the two groups. However, in the CCRT + AC group, 4 patients (3.3%) had grades 3–4 hear loss, and 2 (1.6%) had grades 3–4 TLI.

**Table 3:** Treatment-related toxicities in patients with locoregionally advanced NPC treated with NAC plus IMRT vs. CCRT plus AC

Toxicity	NAC + IMRT [cases (%)]	CCRT + AC [cases (%)]	*χ* ^2^	*P*
Grade 3/4 acute toxicities				
Leukopenia	1 (0.9)	12 (9.8)	7.618	0.006
Anemia	0	3 (2.4)	1.252	0.263
Thrombocytopenia	1 (0.9)	4 (3.3)	0.719	0.397
Hepatotoxicity	0	1 (0.8)	0.000	1.000
Nausea–vomiting	2 (1.7)	10 (8.1)	5.204	0.023
Mucositis	5 (4.3)	8 (6.5)	0.582	0.445
Late toxicities				
Skin dystrophy	37 (31.6)	51 (41.5)	2.500	0.114
Subcutaneous fibrosis	26 (22.2)	29 (23.6)	0.062	0.803
Xerostomia	68 (58.1)	85 (69.1)	3.132	0.077
Hear loss	54 (46.2)	64 (52.0)	0.829	0.363
Temporal lobe injury	4 (3.4)	6 (4.9)	0.059	0.809

Abbreviations as in Table 1

### Survival analysis

**Fig. 1:**
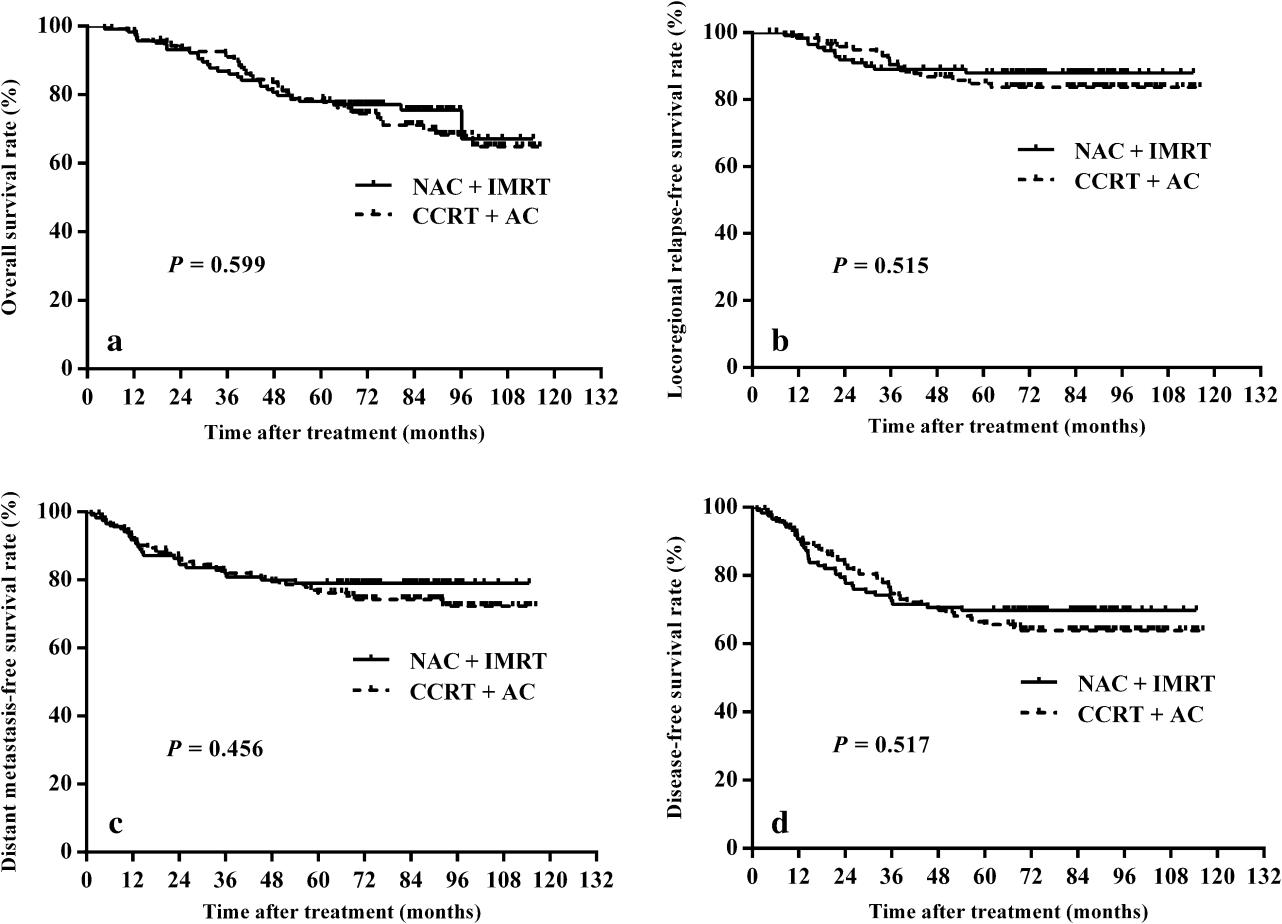
Kaplan–Meier estimates of the survival of patients with locoregionally advanced nasopharyngeal carcinoma (NPC) treated by neoadjuvant chemotherapy (NAC) plus intensity-modulated radiotherapy (IMRT) vs. concurrent chemoradiotherapy (CCRT) plus adjuvant chemotherapy (AC). **a** overall survival; **b** locoregional relapse-free survival; **c** distant metastasis-free survival; **d** disease-free survival. There were no significant differences in survival between the two groups

The 5-year OS, LRRFS, DMFS, and DFS rates were 78.0, 87.9, 79.0, and 69.8%, respectively, for the NAC + IMRT group, and 78.7, 84.8, 76.2, and 65.6%, respectively, for the CCRT + AC group. There were no significant differences in survival between the two groups (Fig. 1). Subset analyses revealed that the differences in OS, LRRFS, DMFS, and DFS were significant in all subsets (Table 4).

**Table 4:** Subset analyses on tumor control in patients with locoregionally advanced NPC treated by NAC plus IMRT vs. CCRT plus AC

Stage	OS		LRRFS		DMFS		DFS	
	HR (95% CI)	*P*	HR (95% CI)	*P*	HR (95% CI)	*P*	HR (95% CI)	*P*
Overall	1.14 (0.70–1.87)	0.599	1.27 (0.62–2.59)	0.515	1.22 (0.72–2.08)	0.456	1.16 (0.74–1.81)	0.517
III	1.52 (0.68–3.39)	0.308	2.23 (0.79–6.24)	0.119	1.62 (0.69–3.80)	0.259	1.62 (0.83–3.16)	0.155
IV	1.04 (0.55–1.96)	0.912	0.65 (0.21–1.98)	0.440	1.11 (0.55–2.21)	0.777	0.92 (0.50–1.70)	0.789
T1–2	0.70 (0.14–3.48)	0.663	50.52 (0–6.22 × 10^8^)	0.386	0.48 (0.11–2.17)	0.332	0.65 (0.16–2.60)	0.537
T3–4	1.23 (0.73–2.07)	0.430	1.25 (0.61–2.57)	0.548	1.41 (0.80–2.49)	0.237	1.27 (0.79–2.02)	0.325
N0–1	1.17 (0.60–2.28)	0.638	1.38 (0.55–3.47)	0.487	1.12 (0.54–2.32)	0.766	1.08 (0.60–1.94)	0.797
N2–3	1.11 (0.52–2.37)	0.783	1.01 (0.31–3.30)	0.990	1.42 (0.66–3.07)	0.370	1.27 (0.64–2.52)	0.487

*OS* overall survival, *LRRFS* locoregional relapse-free survival, *DMFS* distant metastasis-free survival, *DFS* disease-free survival, *HR* hazard ratio, *CI* confidence interval. Other abbreviations as in Table 1

### Prognostic factors

The various potential prognostic factors for predicting LRRFS, DMFS, DFS, and OS rates, including gender, age, WHO histology, T stage, N stage, overall stage, and treatment, were evaluated in univariate and multivariate analysis. In univariate analysis, age and overall stage were significant prognostic factors for OS, DMFS, and DFS (Table 5). In multivariate analysis, age and overall stage were found to be the independent predictors for OS and DFS, and overall stage was a significant prognostic factor for DMFS (Table 6). No significant prognostic factors were found for LRRFS in either univariate or multivariate analysis.

**Table 5:** Univariate analysis of prognostic factors for patients with locoregionally advanced NPC

Variate	5-year survival rate (%)							
	OS	*P*	LRRFS	*P*	DMFS	*P*	DFS	*P*
Gender		0.173		0.587		0.077		0.224
Male	75.8		87.1		75.3		65.9	
Female	88.0		83.3		85.8		74.0	
Age (years)		0.002		0.489		0.025		0.007
<50	82.8		87.0		81.5		73.1	
≥50	68.6		84.6		68.4		55.6	
WHO histology		0.362		0.564		0.526		0.549
Type II	87.1		90.3		83.3		74.5	
Type III	77.5		85.8		76.9		66.9	
T stage		0.213		0.070		0.773		0.299
T1–2	84.4		96.4		81.3		78.1	
T3–4	77.4		84.6		76.9		66.0	
N stage		0.934		0.475		0.414		0.994
N0–1	80.8		85.2		79.4		67.2	
N2–3	75.1		87.8		74.9		68.2	
Overall stage		<0.001		0.518		0.001		0.004
III	85.6		87.7		84.9		74.7	
IV	66.5		83.5		65.3		56.2	
Treatment		0.599		0.515		0.456		0.517
NAC + IMRT	78.0		87.9		79.0		69.8	
CCRT + AC	78.7		84.8		76.2		65.6

**Table 6:** Multivariate analysis of prognostic factors for patients with locoregionally advanced NPC

Variable	OS		LRRFS		DMFS		DFS	
	HR (95% CI)	*P*	HR (95% CI)	*P*	HR (95% CI)	*P*	HR (95% CI)	*P*
Gender (males vs. females)	0.63 (0.32–1.26)	0.191	1.25 (0.55–2.85)	0.588	0.50 (0.22–1.11)	0.087	0.71 (0.39–1.30)	0.265
Age (<50 vs. ≥50 years)	1.92 (1.17–3.15)	0.010	1.31 (0.62–2.77)	0.486	1.59 (0.93–2.72)	0.092	1.70 (1.08–2.67)	0.022
WHO histology (Type II vs. Type III)	1.87 (0.67–5.21)	0.229	1.54 (0.36–6.50)	0.561	1.59 (0.57–4.44)	0.379	1.22 (0.77–1.92)	0.433
T stage (T1–2 vs. T3–4)	1.39 (0.52–3.67)	0.512	5.69 (071–45.81)	0.102	1.00 (0.39–2.59)	0.999	1.36 (0.58–3.19)	0.475
N stage (N0–1 vs. N2–3)	1.36 (0.79–2.37)	0.270	1.13 (0.51–2.47)	0.768	1.59 (0.88–2.87)	0.126	1.29 (0.78–2.13)	0.322
Overall stage (III vs. IV)	2.50 (1.47–4.26)	0.001	1.05 (0.51–2.17)	0.897	2.54 (1.41–4.58)	0.002	1.80 (1.12–2.89)	0.016
Treatment (NAC + IMRT vs. CCRT + AC)	1.23 (0.74–2.03)	0.426	1.34 (0.65–2.77)	0.436	1.35 (0.78–2.32)	0.281	1.40 (0.60–3.25)	0.397

## Discussion

For locoregionally advanced NPC, the present study demonstrated that NAC plus IMRT and CCRT plus AC resulted in similar outcomes in terms of 5-year OS, LRRFS, DMFS, and DFS rates. A subgroup analysis indicated that the effect of concurrent and adjuvant chemotherapy on OS, LRRFS, DMFS, and DFS was insignificant in all subsets.

CCRT has been considered the standard of care for locoregionally advanced NPC [20]. Although the efficacy of chemotherapy delivered concurrently with conventional radiotherapy has been repeatedly proven [4, 6, 21], the most effective combination of chemotherapy and IMRT has not been well established.

Three retrospective studies have evaluated the contribution of chemotherapy for patients with NPC treated with IMRT [22–24]. Lin et al. [22] reported that IMRT following NAC for locoregionally advanced NPC provided a favorable outcome in terms of 3-year local/regional control, metastasis-free survival (MFS), DFS, and OS; furthermore, their results suggested that concurrent chemotherapy offered no significant value for further improvement of local and regional control to IMRT following NAC. Su et al. [23] demonstrated that patients with locoregionally advanced NPC had similar OS, MFS, and DFS when treated with IMRT-based modalities, including IMRT alone, NAC plus IMRT, IMRT plus AC, CCRT alone, NAC plus CCRT, and CCRT plus AC. Another retrospective study assigned 276 patients with locoregionally advanced NPC to compare IMRT alone with NAC plus IMRT, CCRT alone, and NAC plus CCRT [24]. The results revealed that the addition of concurrent or neoadjuvant-concurrent chemotherapy to IMRT prolonged relapse-free survival (RFS) or DFS for patients with locoregionally advanced NPC, whereas NAC provided no significant benefit for OS, MFS, RFS, and DFS.

In the current retrospective cohort study, NAC plus IMRT produced a superb outcome similar to CCRT plus AC in terms of locoregional control in patients with locoregionally advanced NPC (87.9 vs. 84.8%, *P* = 0.515). We hypothesized that the application of IMRT would improve the local control rate, which may “counteract” the effect of CCRT on improving the local control rate and survival rate. In addition, NAC could reduce hypoxia in the primary site and metastatic lymph nodes by shrinking the tumor, which could increase radio-sensitivity and increase locoregional control [25]. After 2 cycles of NAC with cisplatin plus 5-FU, 5 patients (4.3%) had CR, and 93 (79.5%) had PR in the NAC + IMRT group.

Although the use of IMRT has produced significant improvements in LRRFS, effective treatment of distant metastases remains an important problem to be solved. In the present study, 23 patients experienced a local or regional relapse, and 48 developed a distant metastasis. Distant metastasis remained the predominant mode of treatment failure, which was consistent with the results of other reports [26, 27]. Our results demonstrated that NAC plus IMRT achieved similar DMFS compared with CCRT plus AC (79.0 vs. 76.2%, *P* = 0.456). The main goal of NAC is to eradicate distant micrometastases [28]. Given the high distant failure rate associated with NPC, it is logical to expect a decline in distant failure with the use of NAC. A recent meta-analysis that emphasized the use of NAC revealed that NAC could effectively enhance OS and reduce DMR [14]. However, both the drug dose and course necessary to eradicate all distant micrometastases are still unknown. As a result, we recommend further investigation on the optimal regimen of NAC for locoregionally advanced NPC.

A multivariable analysis for prognostic factors was also performed in the present study. OS and DFS were both independently affected by age and overall stage, and DMFS was only affected by overall stage. It is well known that overall stage is undoubtedly the most important prognostic factor for predicting the survival of NPC patients [29, 30].

Despite of the proven efficacy of chemotherapy delivered concurrently with conventional radiation, the combined treatment strategy comes with substantial adverse effects. In the pivotal INT-0099 trial, the proportions of patients who could complete the scheduled concurrent and adjuvant chemotherapy were only 63 and 55%, respectively, due to excess toxicity [4]. Similar results on treatment-related toxicities have been documented in retrospective and randomized studies [6, 21, 31].

In the present study, the group of patients who received CCRT plus AC had significantly more severe leukopenia and nausea–vomiting than patients who received NAC plus IMRT. In the CCRT + AC group, most of the grades 3–4 nausea–vomiting occurred during the CCRT phase. Compared with the CCRT + AC group, the NAC + IMRT group had less gastrointestinal and hematologic toxicities. Studies by Lin et al. [22] and Sun et al. [32] also demonstrated that the total occurrence rates of grade 3 or 4 acute toxicities in patients receiving concurrent chemotherapy was higher than those who received IMRT alone. No significant differences in late toxicities were found between the two groups. In our study, in the NAC + IMRT group, skin dystrophy, subcutaneous fibrosis, xerostomia, and TLI were mild (grades 1–2); however, in the CCRT + AC group, 4 patients (3.3%) had grades 3–4 hear loss, and 2 (1.6%) had grades 3–4 TLI. One possible explanation for milder toxicities in the NAC + IMRT group may be that NAC was likely to reduce primary tumor volume for patients with intracranial invasion, and re-planning of the delineation for tumor volume after NAC could better protect critical normal tissue and reduce IMRT-associated adverse events [33].

These results demonstrated that a regimen of CCRT plus AC that significantly increases the probability and severity of treatment-related adverse events might not be essential for the treatment of NPC if NAC and IMRT are used instead. We hypothesize that omission of concurrent-adjuvant chemotherapy may be possible, and effective disease control in the primary area and neck lymph nodes can be achieved with improvement in radiation technology and use of sequential chemotherapy. Further randomized clinical trials are necessary for the establishment of the most effective combination of chemotherapy and IMRT to improve the prognosis of patients with locoregionally advanced NPC.

## Conclusions

The treatment outcomes of NAC plus IMRT and CCRT plus AC for locoregionally advanced NPC were similar. Distant metastasis remained the predominant mode of treatment failure. The most effective combination of chemotherapy and IMRT needs to be established through further randomized clinical trials.
